# Chapter II: General Concomitants of Head Symptoms

**Published:** 1889

**Authors:** Edmund J. Lee


**Abdomen**, with bearing down in: phyt.
— **heat** of: tarent.
— **pain** in: acon. æth. ailan. *aloe.* bism. cedr. cina. cocc. colch. coloc. con. cupr. dig. dios. euphr. gymn. hepar. hydrs. hyos. indg. iris. lach. led. lyc. merc. naja. nit-ac. nitr. ol-an. phos. ptel. rhus-r. stram. tarent. tereb. verat. verat-v. znc.
— — alternating with: cina.
— — in right iliac region: lil-t.
— — in liver: indm. tarent.
— — in pelvis: stram.
— — in spleen: borax. cinnb. urt-ur.
— — in umbilicus: lil-t.
— **stitches** in hypochondria: æsc-h.
— **swelling** of: nitr. phos. stram.
**Anguish**, with: ailan. anac. ant-c. lach. nat-m. selen. seneg. stann. stram.
**Anxiety**, with: ant-t. benz-ac. cact. cadm. carb-an. chel. chin-s. glon. jabor. nat-c. nitr. phos. plat. ran-b. rheum. ruta. sep. stront. tarent.
**Apathy**, with: bell.
— **aversion** for motion: ph-ac.
— — for speaking: ant-c. rhus-r.
— — for thinking: ailan. equ. phyt. rhus-r. tep.
— — for work: calc-p. camph. coff. coloc. dig. dulc. lact. op. phos. sep.
— **disgust** of life: podo.
**Appetite**, with increase of: chin-s. elaps. iod. lyc. psor. sil.
— with loss of: ailan. anac. benz-ac. calc-p. con. euphr. ferr. gymn. hipp. iber. *lach.* nat-m. petr. phos. selen. sep. stann. stram. tabac.
**Arms**, jerking: verat.
— see also Hands.
— **pain** in: arg-n. bell. bry. calc-p. carb-v. cimic. dios. dirc. euphr. gymn. indg. lach. mag-c. phos. tilia. verat.
— — arms and hands, pain in: calc-p.
— — in elbows: indg. lobel.
— **numbness** in: all-c. phys.
— **tingling** in: ailan.
— **weakness**: glon. iod.
**Asthma**, with: coloc.
**Awkwardness**, with: caps.
**Back**, chilliness of, with: sil.
— **numbness**, in a band, from left scapula to hip: ailan.
— **pain**, with: ailan. benz-ac. cina. cob. daph. fluor-ac. graph. hydrs. meni. merc. myric. ol-an. op. sabad. sabin. *sil.* verat. trios. ziz.
— — in small of: apoc. cob. lac-can. sil.
— — — — alternating with: brom.
— **sweat**, cold, on: acon. morph-ac.
— **weakness** of, with: coloc. fago. lobel. nym.
**Blood**, feels as if arrested: bar. sulph.
— **congestion** of, with. See under Congestion, page 93.
**Breath**, fetid, with: apis.
— **cool**, sense of, with: arg-n.
**Cheeks**, hot, with: calc. cann-s. merc.
— **red**, with: alum. bov. cann-s. *ign.* znc.
— **swelling** of, with: calc. carb-v. lyc.
— see also under Face.
**Cheerfulness**, with: coca. lyc. phos. ph-ac.
**Chest**, constriction of, with: sep.
— **oppression** of, with: ailan. carb-ac. crotal. meli. tarent.
— — — alternating with: glon.
— — — compare with Respiration.
— **pain** in, with: æth. almn. cham. chro-ac. cina. con. dulc. eupi. fago. graph. gymn. hydrs. hydrph. indg. jabor. k-ca. *lach.* lith. merc-i-r. nat-m. op. ran-b. stront. tarent. tep.
— — alternating with: iod.
— **sinking** sensation in, with: nx-v.
— see also under Respiration.
**Chilliness**, with: aloe. anac. arg. arg-n. arn. calc. cadm. camph. carb-v. chin. con. dios. dulc. eup-per. euphr. evon. ferr. hell. hyper. indg. indm. jatr. k-iod. lach. lam. mag-s. mang. meni. mezer. nitr. nit-ac. puls. rhus. sang. sep. sil. stann. sulph. tarent. thu.
— after headache: sang.
**Coition**, desire for: sep.
**Coma**, with: chin-s.
**Confusion**, mental, with: *aur.* glon. nat-ars. petr. stram.
— — as if would lose senses or go mad: *acon.* agar. chin. stram. tarent. verat.
— unable to collect one's senses: carb-v. chin. creos. crotal. cyc. *mang.* mezer. nit-ac. rhus. sars stann. sulph.
— see also Confusion, Stupefaction under Mind, Head.

**Constipation**, with: aloe. alum. bry. coff. con. crotal. ign. lach. mag-c. merc. nx-v. op. petr. puls. verat. znc.
**Coryza**, with: *acon.* æsc-h. all-c. arg-n. ars. bad. *bell.* bov. bry. calc. carb-v. caus. cham. *chin.* cina. cic. coral. croc. dios. euphr. ferr. hell. hepar. ign. jac. k-ca. k-iod. lach. lyc. mag-m. merc. nit-ac. *nx-v.* phos. phyt. psor. puls. rhod. sabad. sep. spig. thu.
— with dry: croc.
**Cries**, extorting: cact. cimic. coloc. cupr. hydrph. k-ca. mag-m. petr. sep. sil. stann. tarent. tong.
**Delirium**, with: chin-s. sil. stram.
**Delusions**, with: chin.
**Depression**, dejection, with: agar. anac. aur. berb. cact. con. creos. crotal. dulc. iris. lac-can. lact. mag-c. naja. ran-b. rhus-r. selen. sep. sil. tanac. tarent. therid.
**Despair**, with: agar. tarent. vip-red.
**Diarrhœa**, with: æth. agar. aloe. ambr. apis. con. glon. graph. indm. jatr. nitr. stram. verat.
— alternating with: podo.
**Dizziness.** See Giddiness.
**Dreams**, with: alum. aur-m. borax. chin-s. puls.
**Ears**, **discharge**, from, with: borax. psor.
— **hearing**, hardness of, with: arn. dulc. grat. ign. lyc. stram.
— — improved: phyt.
— **heat** of, with: calc. gran. lyc. mur-ac.
— — right hot, left cold: tereb.
— **noises** in, with: acon. anac. ars. borax. bry. chel. chin. *chin-s.* clem. coca. cocc. creos. dulc. euphr. ferr. gels. hyper. lach. lyc. murx. myric. nicc. op. petr. ph-ac. plat. *puls.* rhus. sang. selen. squil. staph. sulph. tarent. thu. verat.
— — gurgling in: plat.
— — hammering in: spig.
— **pain** in, with: agar. all-c. alum. anac. arn. arum-t. asar. bar. bell. borax. bov. brom. cact. calc. canth. caps. carb-an. caus. chel. cimic. colch. con. glon. gymn. ham. hura. ign. indg. k-bi. kalm. lach. lepi. lil-t. lith. lyc. mang. merc. merc-i-r. mosch. nat-m. nitr. ol-an. plb. prun. puls. ran-sc. rhus. sang. sars. sep. sol-n. sulph. tabac. tarent. tilia. tong. viol-od. znc.
— **stitches** in, with: borax. croton. merc. rhus.
— **stoppage** of, with: merc-i-fl. selen.
— **tickling** in, with: borax. mur-ac.
**Epileptic** attack, after an: cina. cupr.
**Epistaxis**, with: agar. alum. ant-c. berb. bry. cadm. carb-an. coff. crotal. dulc. ferr. ham. lach. sep. tep.
**Eructations**, with: arg-n. bism. calc. carb-v. chin-s. cyc. graph. iod. lach. lyc. nat-c. nit-ac. nx-v. op. phos. phys. psor. sil. sulph. uran.
**Excitement**, with: bapt. chin. phos. ruta.
**Eyes**, bloatedness of, with: lach. rheum.
— **blindness**, with: ether. grat. iris. k-bi. petr. znc.
— — followed by headache, sight returns with increasing headache: k-bi.
— **closing** of lids, with: agar. aloe. am-c. ant-t. arg-n. bell. calc. carb an. carb-v. cedr. cocc. creos. lith. mag-c. mezer. mosch. nat-m. nit-ac. nitr. nx-m. nx-v. olnd. petr. ph-ac. plat. plb. sep. sulph.
— — with pain in forehead: agar. am-c. ant-t. bell. cann-s. carb-v. cedr. chel. coff. coral. eupi. gels. glon. lact-ac. nat-s. nit-ac. nx-m. op. phos. plat. podo. sep.
— — must close: canth. coral. stram.
— **congestion**, with: alum. bell. myric. nat-m. stram.
— **diplopia**: con. kiss.
— **downward** pressure, with: carb-an.
— **drawing** in lids, with: acon. bell. creos.
— **fall** out, sensation as if would: brom. glon. nitr. phos. rhus. sang. sep. valer.
— **flashes** before: chin-s. coca. viol-od.
— **flickering** before: chin-s. con. cyc. lach. phos. sars.
— **heat** in, with: ailan. ambr. bov. eugen. eupi. gymn. lyc. tarent.
— **heaviness** in, with: aloe. arum-t. coca. ham. peti.
— **inflamed**, with: bad. calc-s. led. merc-i-r.
— **dim vision**, with: am-c. arg. aster. bov. bry. carb-an. croc. cyc. ferr. gels. glon. grat. helon. hydrs. *hyos.* ign. indg. jug-c. k-bi. k-ca. lil-t. mag-m. meph. mur-ac. nat-c. nat-m. nit-ac.

nx-v. petr. phos. *puls.* raph. sars. sep. sil. stann. *stram.* sulph. thu. tilia. znc.
— — sees half light, half dark: *glon.*
— **lachrymation**, with: agar. arg-n. asar. bell. bov. carb-an. carb-v. chel. comoc. con. eugen. *ign.* indm. k-iod. lac-can. lil-t. merc. osm. plat. *puls.* rhus-r. spong. stram. tax.
— **motion** to side, difficult: mag-s.
— **open**, difficult to: nat-m. op. peti. ph-ac. plect. stram.
— **pain** in, with: acon. ambr. anac. ant-t. arg-n. ars. *bar. bell.* bism. *bry.* calc. calc-p. carb-v. caus. chin-s. cic. cimic. cina. *cocc.* con. creos. croc. croton. eugen. hipp. ign. k-ca. lach. led. lyc. mag-c. mag-m. merc. mezer. mosch. mur-ac. nat-c. nicc. nit-ac. nx-v. op. par. petr. phos. psor. *puls.* ran-b. rhod. rhus. sabad. sabin. samb. seneg. sep. *sil.* spig. spong. stann. stram. stront. sulph. valer. znc.
— — with the pain the eyes become smaller: aloe.
— — in orbits: acon. aloe. bad. bar-ac. bell. calc. camph. carb-an. chin. clem. con. crotal. hydr-ac. ign. lach. lil-t. lith. mang. merc-i-fl. nat-ars. nuph. op. pæon. seneg. stront. sulph. tabac. znc.
— — see also Aching, Forehead, page 78.
— **photophobia**, with: arg-n. glon. ign. k-bi. k-ca. nat-s. stram. sulph. tarent.
— — compare with Closing of Eyes.
— **pupils**, dilated, with: acon. bell. laur. morp-ac. rheum. verat-v.
— — contracted with: bell. laur. sep. sol-n. verat.
— **redness** of, with: arg. arg-n. bell. cinnb. cupr.
— **sparks** before, with: chin-s. coca. cyc. eugen. lach. phos. psor. sars. spong. viol-od.
— **spasm** of, with: viol-od.
— **stiff** sensation in, with: mag-s. nit-ac.
— **stinging** in, with: puls.
— **swelling** of, with: hyos. lach. rheum.
— — sensation as of: meni.
— **twitching** of lids, with: coloc. creos. mill. mur-ac.
— **yellowness** of: myric.
— — objects appear in a yellow veil: k-bi.

**Face, coldness** of, with: rhod.
— **distorted**, with: graph.
— **erysipelas** of, with hammering headache: lach.
— **heat of**, with: *acon.* agar. aloe. ang. aran-d. arn. asaf. bry. calc. calc-p. cann-s. canth. chin-s. coff. creos. ferr. gels. glon. grat. k-ca. laur. lobel. mag-c. men. nat-m. nx-v. op. phos. plat. psor. ran-b. rhus. sabad. sil. stront. sulph. tarax.
— **numbness** of, with: bapt.
— **pain** in, with: acon. agar. ambr. am-m. arg-n. berb. bry. calc. carb-an. carb-v. chel. cina. clem. colch. creos. crotal. dros. euph. evon. graph. hepar. lach. laur. lil-t. lyc. mosch. nat-m. nitr. nx-m. *nx-v.* osm. petr. phos. puls. rhus. seneg. sil. spig. spong. stront. sulph. tarax. thu. tong. urt-ur. viol-od.
— **paleness** of, with: acon. alum. ambr. canth. chin-s. hell. hydrs. lach. mag-c. phos. spig. valer. *verat.* znc.
— **redness** of, with: acon. ailan. *bell.* bov. bry. cact. calc. camph. cann-s. canth. coff. creos. ferr. glon. hydrph. ign. indg. indm. ipec. k-iod. kalm. lach. lyc. mag-c. mag-m. morp-ac. nat-c. nat-m. *nx-v. op.* phos. plat. plb. rhus. sil. spong. stront. sulph. tarax. thu. znc.
— **sweat** on, with: glon. mag-c. morp-ac. plat. thu.
— **swelling** of, with: acon. ailan. calc. k-bi. lach.
— **yellowness** of, with: lachn. vip-red.

**Faintness**, with: berb. calc. graph. lach. lyc. merc-i-fl. nat-m. nx-v. petr. puls. sil. stram. vip-red.

**Fear**, with: *acon.* ambr. fluor-ac. glon. stront. tereb.

**Feet, cold**, with: bufo. camph. chro-ac. coca. ferr. men. naja. stram. sulph. trios.
— **heaviness** of, with: clem.
— **pain** in, with: sang. stront.
— — in toes: fluor-ac. op.
— **stiffness** of, with: trios.
— **weariness** of, with: carb-an.
— see also under Limbs.

**Fever**, with: ars. chin-s. ferr. hell. helon. hydrph. indm. lach. phos. rhod.
— with evening: led. lobel.
— see also Heat.

**Fingers**, coldness of, with : hell.
— **paleness** of, with : verat.
— **pains** in, with: calc-p. cyc. nitr.
— — in thumb : indg.
— see also under Hand.
**Flatulency**, with : calc-p. carb-v. chin-s. ferr. glon. led. naja. rumx.
**Forgetfulness**, with : calc-ac. caps. chin-s. mezer. sars.
**Fretfulness**, with: k-ca. k-iod. sil. stann. thu. tong.
**Giddiness**, vertigo, etc., with: acon. *æth.* agar. ailan. alum. anac. ang. ant-t. apis. arg. arg-n. arn. asar. aur. bar. bell. berb. bism. brom. *bry. calc.* canth. carb-an. caus. cham. chin. chin-s. clem. coca. cocc. con. creos. crotal. cupr. cyc. dig. dios. dulc. euph. ferr. fluor-ac. gels. glon. graph. grat. ham. hell. helon. hepar. hipp. hydr-ac. hyos. ign. iris. k-bi. k-clc. kalm. *lach.* lam. laur. lil-t. lobel. lyc. mag-c. mag-m. mag-s. meph. merc. mezer. mur-ac. nat-c. nat-m. nicc. nitr. nit-ac. nx-j. nx-m. *nx-v.* ol-an. op. petr. *phos.* phyt. podo. prun. psor. *puls.* ran-b. rheum. rhus. sabad. samb. sang. secale. sep. spig. stann. stram. stront. sulph. tabac. tarax. verat. verb. znc.
— after headache : merc-s.
**Glands**, pain in cervical, with: am-c. borax. bry.
— — parotid : bry. k-bi. sabad.
— **swelling** of cervical: bar. mur-ac.
**Grimaces**, makes: agar.
**Groaning**, moaning, with: ars. bell. sil.
**Hair**, falling out, after repeated attacks of headache: hepar.
**Hands**, with cold : ambr. benz-ac. borax. camph. indm. lact. men. merc. ran-b. stram.
— **heat** of, with: ign. lact. phel.
— — alternately hot and cold: borax.
— **numbness** of, with: hydrph.
— **pain** in, with: calc-p. phos. tarent.
— **paralyzed** feeling in, with : lobel.
— see also under Fingers, Limbs, etc.
**Heart**, anxiety at: plat.
— **fluttering** sensation : form.
— **pains** at: hydrph. jab. lycps. merc-i-r. seneg. sulph. tarent.
— **palpitation** of: acon. alum. ant-t. bufo. clem. hepar. elect. jab. lach. nx-v. spig.
— — with each throb of: bufo. *cimic. glon.* ign.
— **sinking** at: arn.
— **uneasiness** at: dig.
— **violent** action of: glon. lycps.
**Heat**, with ; æth. agar. ailan. alum. ang. arg. arg-n. bism. bov. calc. camph. chin. coral. creos. cyc. dig. gamb. glon. hepar. hyos. indm. lach. lyc. mag-s. merc. morp-ac. nat-m. nat-s. nitr. nx-v. oxal-ac. plat. puls. rhus. sang. sulph. tarent. tilia. vip-red.
— alternately hot and cold: borax. carb-v.
— — of rigor and heat: berb.
**Humming** in head, with: puls. rhus.
**Hurry**. See Impatience.
**Ill-humor**, with : am-c. am-m. anac. bell. bov. calc-p. chin-s. coloc. con. creos. dulc. indm. k-ca. mag-m. merc. nat-m. nicc. op. pæon. phos. plat. sil. stann. stram.
**Impatience**, with: plat. ptel. rhus-r. znc.
**Indifference**, with: nat-ars. op. puls. tarent.
**Indolence**, with: calc-p. dulc. lact. laur. ph-ac.
— see also Apathy.
**Irritability**, with: am-c. am-m. anac. bell. berb. bov. calc-p. chin. coloc. con. creos. dulc. k-ca. k-iod. kalm. lac-can. laur. mag-m. meph. merc. nat-m. nicc. nx-v. op. phos. *plat.* sil. stann. thu. tong.
**Jaws, pain** in, with: ang. aral. brom. bry. calc. canth. carb-v. cimic. con. gels. hura. hydrph. k-clc. lach. merc-i-fl. mezer. nat-m. nit-ac. nx-v. par. plat. ran-b. stict. sulph. valer. vip.
— — **in upper** : acon. ant-t. chin. creos. k-bi. stront. sulph. thu.
— — **in lower**: ang. arum-t. bar-ac. brom. *canth.* carb-v. dios. hura. indg. lach. mang. nit-ac. oxal-ac. par. plat. ran-b. stict. sulph. tarax.
— numbness of: hura.
— swelling of lower: acon.
— tremor of: carb-v.
**Lay the head** on something, must: con.
**Laughter**, inclination to, with: sabad.
**Lie down**, must: alum. am-c. anac. bell. bry. *calc.* calc-p. chin. *con.* crotal. euphr. ferr. graph. iod. k-bi. k-ca. lach. lyc. mag-m. mosch. nat-c. nat-m. nit-ac. *nx-v.* olnd. op. petr. ph-ac. puls. rhus. *sang.* sars. *selen. sep.* sil. stann. sulph. znc.

— — unable to: coloc.
— — compare with Lying, relieves.
**Limbs, aching** in joints, with: erig.
— **coldness** of, with: camph. k-ca. sulph.
— **cramp** in calves: cann-i.
— **heaviness** of, with: *carb-v.* nit-ac. sabad. sil.
— **pain** in, with: acon. ailan. apoc. cact. carb-v. coll. erig. gels. lach. mag-c. *sang.* sulph.
— — in legs: agar. calc. calc-p. cedr. crotal. gymn. k-ca. kalm. mag-c. morp-ac. osm.
— — in hip-joint: nitr.
— — in knee: oxac-ac.
— **trembling** of, with: lyc. sulph.
— **weakness** of, with: ant-c. glon. k-bi.
— — of legs: castor. nx-v.
— compare Arms, Hands, Feet, etc.
**Mania**, with: cocc. iod.
— with fear of: ambr.
**Memory**, lost, with: con.
— see Confusion, etc.
**Mental** powers, diminished, with: ailan. aster. aur. bov. cann-i. cann-s. chel. creos. cyc. glon. hell. hyos. ign. kalm. laur. nx-m. op. prun. rhus. znc.
— — — loss of thoughts: asar. cocc. creos.
**Mind**, loss of, with: *acon.* ambr. cocc. mag-c.
**Menses**, too soon, with: carb-v. gent. ol-an.
— — — and too scanty: alum. nat-m.
— — — and too profuse: laur.
— **profuse**, with: mag-c. sang.
— — and too late: nit-ac.
— irregular and watery, with: berb.
— see also on page 148.
**Moaning**. See Groaning.
**Motion**, averse to: ph-ac.
**Mouth**, burning of, with: k-iod.
— **dryness** of, with: dios. lyc. naja. puls.
— — of lips: cod. indm. nx-v. rhus. ziz.
— **heat** of, with: carb-v.
— **pain** in palate, with: merc. nat-m.
— — in gums: daph.
— **slimy**: bell.
— **swelling** of gums, with: hura.
— see also Jaws, Teeth, Tongue, etc.
**Nausea**, with: acon. æsc-h. ailan. *alum.* *am-c.* *ant-c.* apis. arg. arg-n. arn. ars. asar. aur. benz-ac. borax. *bry.* calad. *calc.* camph. cann-s. caps. *carb-v.* caus. chin. chin-s. cic. cocc. *coloc.* con. coral. croc. crotal. dros. dulc. *eugen.* eup-per. eup-pur. fluor-ac. glon. graph. grat. hepar. ign. *ipec.* *iris.* k-bi. k-ca. lac-can. *lach.* lyc. mag-c. meph. merc. *mosch.* nat-c. *nat-m.* nit-ac. *nx-v.* petr. phos. phyt. plat. *puls.* ran-b. rhus. ruta. *sang.* sars. seneg. *sep.* sil. spig. *stann.* stram. stront. *sulph.* *tabac.* tarax. therid. *verat.* znc. etc.
— — headache aggr. while nausea lasted: petr.
— with inclination to vomit: *alum.* arg. ars. benz-ac. *calc.* camph. cocc. con. grat. ign. indg. sep. tabac., etc.
— — see also Eructation.
— **with vomiting**: æth. agn. apis. arg-n. arn. bar-m. bry. cadm. caps. caus. chin. cimx. cocc. coloc. con. creos. croton. dulc. eugen. ferr. glon. graph. ipec. iris. jatr. k-bi. k-ca. lach. mezer. mosch. nat-m. nit-ac. nx-m. *nx-v.* op. phos. plat. *puls.* rhus-r. *sang.* sars. *sep.* sil. spig. stann. stram. *tabac.* verat. verat-v. vip. znc.
— — — of bile: arg-n. aur. croton. iris. sang. znc. ziz.
— — — sour: apis. nx-v. op. sars.
— — — yellow-green mucus: verat.
— — — yellow bitter mucus: form. glon.
**Neck**, numbness of: spig.
— **pain** in, with: acon. ailan. alum. anac. arg-n. arn. asar. bar. bell. borax. bry. bufo. calc-p. cann-i. cann-s. canth. carb-v. caus. chel. chin. clem. con. elaps. euph. fago. gal-ac. gels. glon. graph. hura. hydr-ac. hydrph. hyos. jac. k-ca. k-iod. kalm. lach. laur. lepi. lil-t. lyc. manc. mag-c. mag-s. merc. merc-i-fl. mosch. myric. peti. plb. ptel. nat-m. ran-b. rhus-r. sars. serp. sulph. spong. tep. ziz.
— — in **nape** of, with: æth. alum. ambr. am-c. anac. asar. bar. bell. berb. borax. bry. cann-s. carb-an. carb-v. caus. chel. cinnb. clem. cocc. con. corn. croton. glon. graph. hell. hyos. hydr-ac. iod. ipec. k-ca. kalm. lil-t. lyc. mag-c. merc. mezer. mosch. mur-ac. nat-c. nat-m. nitr. op. pæon. plect. plb. puls. ran-b. rhus-r. sabin. sars. sil. spong. sulph. tarax. tarent. trios. verat.

— — — when raising the head : senna.
— **stiffness** of, with : am-c. ant-c. arg. bar. calc. caus. crotal. cyc. glon. *graph.* k-ca. lach. mag-c. merc-i-fl. mur-ac. nat-c. *nitr.* ph-ac. sang. sep. spig. tarent. verat.
— **strangulation**, feeling of: glon.
— **weariness** of, with feeling of: fago.
**Noise**, sensitive to : calc. k-bi.
— see also page 149.
**Nose**, heat of, with : conv.
— **itching** in, with : ferr.
— **pain** in, with : acon. agar. ant-t. bism. calc. camph. cic. colch. corn. croc. glon. guiac. hell. ign. lach. *lyc.* merc. merc-i-fl. merc-i-r. mezer. mosch. nat-c. nit-ac. nitr. nx-v. op. phos. ph-ac. plat. ran-b. squil. stann. viol-od.
— — — as if it would bleed : tarent.
— — forehead and nose : agar. bism. brom. croc. colch. dulc. ferr. glon. hipp. ign. k-iod. kiss. lach. lachn. lyc. merc-i-fl. mezer. mosch. nat-c. nitr. nx-j. op. phos. ph-ac. pip-m. plat. ran-b. squil. sulph. tarent. thu.
— — — compare root of nose, under Forehead, page 79.
— **stopped**, with : *acon.* bov. chel. chin. grat. indm. *k-bi.* k-ca. nitr. sulph.
— — see also Coryza, Catarrhal, etc.
**Numbness** of head and face : bapt.
**Olfactory** nerves, with sensitiveness of: phos.
**Pains** all over body, with : op.
— in other parts : lyc.
— in bones : lac-ac.
— gouty pains : sulph.
— rheumatic and stiffness : sang.
— soreness of body : bad.
— see also Arms, Hands, Limbs, etc.
**Perspiration**, with : ant-c. apis. arg. arn. ars. canth. caus. chin-s. glon. hyos. lachn. lyc. mag-s. nat-s. nitr. op. oxal-ac. plat. puls. tarent.
— cold : graph.
— on face : mag-c. plat. thu.
— on head : acon.
— on forehead, warm : glon.
— — — cold : verat.
— precedes the headache : ferr.
— see also Sweat, page 154.
**Pulse, contracted**, with : bism.
— **hard**, with : aster. lach.
— **quick** : asar. chin-s. glon. laur. phos.
— **slow** : canth. chin-s. laur. lyc. phys. sol-n.
— **weak** : ars. peti. vip-red.
**Pyrosis**, with : am-c. lach. op.
**Respiration**, difficult, with : ailan. ars. cact. carb-ac. carb-v. con. crotal. dulc. jab. lact. nit-ac. nitr. sep.
— with disease of respiratory organs : *lact.*
— see also under Chest, Heart, etc.
**Restlessness**, with : anac. arg-n. *ars.* bell. bry. cadm. calad. camph. cham. chin. coral. cyc. daph. dulc. gent. hydrph. ign. indm. k-iod. *lach. lyc.* meni. morp-ac. naja. nitr. nx-m. par. prun. ran-b. ruta. sil. tarent. vip-red.
— with impulse to run hither and thither : ars. coloc.
**Retching.** See Eructation, Nausea, etc.
**Sadness.** See Depression.
**Saliva**, increased, with : ant-c. cinnb. hipp. indm. k-bi. op. phos. sep. verat.
— bloody, with : mag-c.
**Shoulders**, pain in, with : bry. chin. cupr. fluor-ac. gels. ipec. kalm. lach. lac-can. nitr.
— — joint, right side : lach.
— — extends to : bry. gels. glon. graph. ipec. kalm. podo.
**Shuddering**, with : berb. evon. hell. lach. mag-s. mezer. nx-v. puls. sil. thu.
**Sing**, inclined to : nat-m.
**Sleepiness**, with : acon. æsc-h. agar. ailan. ars. asar. bruc. camph. cham. chin-s. con. corn. creos. equ. gels. gins. grat. hipp. hydrs. ign. indg. indm. ipec. *lach.* laur. lobel. merc-i-r. mur-ac. myric. nat-s. nitr. nx-j. *nx-m.* op. phos. puls. ran-b. *stann.* stront. sul-ac. tanac. vip-red. znc.
**Sleeplessness**, with : acon. am-c. bufo. caus. chin. coca. corn. creos. elaps. gent. lyc. merc-i-r. naja. nit-ac. ol-an. par. rhus-r. verat.
**Sneezing**, with : arg-n. arn. gels. k-iod. lach. sulph.
— ineffectual attempts : znc.
**Solitude**, with desire for : ant-c.
**Spasms**, with : petr. stram. verat. vip.
**Speech**, hurried, with : *lach.*
**Spleen**, pain in, with : borax.

**Staring**, with: spig.
**Stomach**, burning in: jatr. sang.
— **pain** in: æsc-h. am-c. apis. arg. ars. benz-ac. casc. clem. cob. dulc. ferr. glon. ham. lith. lyc. mang. naja. phyt. rhus-r. sang. sulph. verat.
— — at pit of: arg.
— see also under Abdomen.
— **empty** sensation in: ham. sang. tax.
— **deranged**. See Nausea.
**Stool**, with urging to: cob. coca. coloc. con.
— see also Diarrhœa.
**Stupefaction**, with: æth. agar. ant-t. arn. carb-v. chin-s. cina. con. cyc. glon. hydrs. k-bi. lyc. mag-c. morp-ac. mosch. nat-m. nx-m. op. phos. ph-ac. sabin. sil. tarax.
**Sweat**. See Perspiration.
**Taciturnity**, with: ant-c. arg-n. con. rhus-r. thu.
**Tænia**: sabad.
**Taste**, altered, with: ailan. calc-p. cyc. lach. morp-ac. op. tarent.
— **bitter**: creos. morp-ac. tarent.
**Teeth**, **chattering** of, with: cadm. carb-v.
— **clinching** of: arg-n. crotal.
— **grating** of: stram.
— **pain** in, with: arg-n. bell. borax. brom. bry. calc. canth. carb-v. castor. caus. coloc. con. creos. croc. crotal. cyc. dig. euphr. fluor-ac. glon. graph. hura. ign. indm. iod. k-bi. kalm. *lach.* lachn. laur. lil-t. lobel. *lyc.* mag-c. merc. mezer. mosch. nat-c. nat-m. nit-ac. petr. prun. psor. *puls.* *rhus.* sabad. sang. sars. sep. stram. sulph. tong. verb. znc.
**Thinking**, averse to: ailan. equ. phyt. rhus-r. tep.
— is difficult: men.
**Thirst**, with: agar. arn. cadm. cham. chin. chin-s. cupr. cyc. eugen. hipp. indm. lach. led. lyc. mang. nat-c. nicc. plat. ran-sc. rhus. stram. tarent. thu. viol-t. vip-red.
**Throat**, **burning** in, with: carb-ac. k-iod. lyc.
— — in œsophagus: der.
— **choking** sensation: ferr.
— **constriction**: cadm. lac-ac. uran.
— **dysphagia**: nitr.
— **dryness**: agar. cimic. coca. naja. plat. stram.
— **pain** in, with: calc-p. croc. jab. lac-ac. merc-i-r. nitr. osm. plat. tarent. verat.
— **soreness** in: agar. podo. stram. tarent.
— **tearing** in: graph.
— **tickling** in: sang.
**Tobacco**, with aversion to: op.
**Tongue**, biting the: agar.
— **dryness** of: nat-m. tarent.
— — and slimy: calc.
— **pain** in: ipec.
— **paralyzed**: lach.
**Tremor**, trembling: acon. agn. arg-n. borax. *calc.* camph. carb-v. chin-s. lact. lyc. op. petr. sars. sep. sulph. tarent.
**Unconsciousness**, with: acon. ambr. arg-n. arn. aur. bell. carb-v. cocc. cyc. glon. k-ca. laur. mag-c. mang. mosch. nat-m. nx-v. petr. prun. puls. rhus. sabin. sil. stram. tarax.
**Urine**, with increased discharge of: acon. canth. cinnb. eugen. *gels.* glon. iris. ol-an. *selen.* uran. verat. vip-red.
— — passes off with profuse discharge of pale urine: *gels.* ign.
**Vertigo**. See Giddiness.
**Voice**, feeble: nat-c. nx-v.
— difficulty in speaking, with: acon. ph-ac. plat.
— hoarse: osm.
— low voice: bell.
— slow speech: cann-i.
**Vomiting**. See under Nausea.
**Weakness**, with: agar. alum. ant-c. arn. ars. bov. chin. chin-s. creos. cupr-ac. cyc. glon. grat. hydrs. hydr-ac. hyos. iod. iris. lil-t. lobel. mag-m. nat-m. nitr. nx-v. ol-an. phos. psor. ran-b. rhus-r. sil. sulph. tarent.
— followed by: lyc.
**Weariness**, with: agar. arn. bell. berb. chin-s. coca. creos. glon. indg. indm. k-ca. lil-t. naja. nat-m. phos.
**Weep**, disposition to, with: ars. carb-v. *coloc.* cop. creos. ferr. phos. *plat.* puls. ran-b. sep.
— see also Cries, Groaning.
**Work**, with aversion to: camph. coff. coloc. dig. phos. sep.
— with inclination for: aloe.
**Wrinkling** of forehead, with: grat. sulph. viol-od.
**Yawning**, with: am-c. chin-s. cyc. ol-an. staph. sulph. znc.

